# The Effect of Ambient Temperature, Relative Humidity, and Temperature–Humidity Index on Stress Hormone and Inflammatory Response in Exercising Adult Standardbred Horses

**DOI:** 10.3390/ani15101436

**Published:** 2025-05-15

**Authors:** Francesca Arfuso, Maria Rizzo, Laura Perillo, Federica Arrigo, Elisabetta Giudice, Giuseppe Piccione, Caterina Faggio, Vincenzo Monteverde

**Affiliations:** 1 Department of Veterinary Sciences, University of Messina, Viale Giovanni Palatucci snc, 98168 Messina, Italy; rizzom@unime.it (M.R.); federica.arrigo@studenti.unime.it (F.A.); egiudice@unime.it (E.G.); gpiccione@unime.it (G.P.); 2 Department of Veterinary Prevention, Provincial Health Company of Palermo, Via Carmelo Onorato, 6, 90129 Palermo, Italy; laura.perillo@asppalermo.org; 3 Department of Chemical, Biological, Pharmaceutical and Environmental Sciences, University of Messina, 98166 Messina, Italy; cfaggio@unime.it; 4 Zooprophylactic Institute of Sicily “A. Mirri”, 90129 Palermo, Italy; vincenzo.monteverde@izssicilia.it

**Keywords:** cortisol, exercise, hematology, heat stress, horse, serum globulins, temperature–humidity index

## Abstract

In the present study, the changes in the markers of stress and inflammatory responses of 12 Standardbred horses were investigated before and after exercise during late spring and summer. The environmental temperature, relative humidity, and temperature–humidity index (THI) were assessed during this study. From horses, blood sampling, measurement of heart and respiratory rate (HR and RR), and measurement of rectal temperature (RT) were performed before exercise (Pre) and within 5 min following the end of the exercise (Post). THI values showed mild stress in June and high stress in July. Direct erythrocyte indices statistically increased after exercise compared to the rest condition (*p* < 0.05). Higher serum cortisol, α1-, α2-, and β-globulin values were found at Post than at Pre (*p* < 0.05), whereas lower serum albumin and A/G ratio values were found at Post than at Pre. The HR, RR, and RT statistically increased at Post than at Pre (*p* < 0.05). The month influenced the values of RR, platelets, cortisol, albumin, α1-, α2-, and β-globulins (*p* < 0.05). Overall, the results of this study confirmed the well-recognized physiological response of athletic horses to re-establish homeostasis following exercise.

## Simple Summary

Humidity and the temperature–humidity index (THI) were monitored throughout this study. Blood samples, heart rate (HR), respiratory rate (RR), and rectal temperature (RT) were recorded before exercise (Pre) and within five minutes post-exercise (Post). THI indicated mild stress in June and high stress in July. Exercise significantly increased direct erythrocyte indices, HR, RR, and RT (*p* < 0.05). Post-exercise, serum cortisol, α1-, α2-, and β-globulin levels were higher, while albumin and the A/G ratio were lower (*p* < 0.05). Monthly variations affected RR, platelets, cortisol, albumin, and globulin fractions (*p* < 0.05). These findings confirm the expected physiological responses in athletic horses to restore homeostasis after exercise.

## 1. Introduction

Horses subjected to physical exercise must promptly respond to the physiological stress that they encounter during training and/or exercise in order to re-establish a new balance. Several body systems, including cardiac, respiratory, musculoskeletal, and endocrine systems in exercising horses work in a coordinated effort through a cascade of normal physiological events to restore and maintain homeostasis [1,2,3,4,5]. This dynamic reaction in exercising horses can be compromised by adverse external factors, such as environmental variables, including environmental temperature and relative humidity outside the comfort zone [6,7,8,9]. In this regard, heat stress can particularly affect well-being, especially in relation to transport and disciplines such as horse riding. Hot and humid climatic conditions can represent stressful circumstances and can cause problems for animal health, leading to illness and injury [6,7,8,9]. The activation of the hypothalamic-pituitary-adrenal axis (HPA), as well as the onset of the acute phase response, is known to be stimulated by a stressful event [6,7,8,9,10]. The HPA and acute phase protein response activation lead to the production of specific hormones (i.e., cortisol) and proteins (i.e., acute phase proteins), which fall within the cascade of reactions involved in the acute stress and inflammatory response [6,7,8,9,10]. The assessment of serum concentration of cortisol and acute phase proteins, together with the hematological profile and physiological parameters (i.e., respiratory rate, heart rate, and rectal temperature) represents a crucial tool to assess the welfare of animals [11,12].

The investigation of the physiological parameters (i.e., respiratory rate, heart rate, and rectal temperature), serum cortisol concentration, and acute phase protein concentration of exercising horses in relation to environmental conditions, such as temperature and relative humidity, allows veterinarians to assess welfare and also communicate feedback to owners and breeders [9,11,12]. The aim of the present study was to assess changes in classical physiological indicators of well-being, as well as markers of stress and inflammatory responses (i.e., heart rate, respiratory rate, rectal temperature, hematological parameters, serum cortisol, serum total protein, and their fractions) as a result of the work performed by Standardbred horses in Sicily (Italy) during the late spring and summer seasons.

## 2. Materials and Methods

### 2.1. Animals and Study Design

A protocol of animal husbandry and experimentation was reviewed and approved in accordance with the standards recommended by the Guide for the Care and Use of Laboratory Animals and Directive 2010/63/EU for animal experiments.

This study enrolled 12 client-owned Standardbred horses (eight mares and four geldings) with an average BCS of 4.83 ± 0.39 and body weight of 459 ± 38 kg. The horses ranged in age from 4 to 8 years and were deemed to be healthy based on clinical examination. This study was conducted during the months of May, June, and July 2022. All animals were stabled in individual boxes (3.5 × 3.5 m^2^) at the same training center located in Sicily, Italy (38°09′07.3″ N, 13°20′47.7″ E, 10 m above sea level), under a natural photoperiod. Horses were fed twice a day (06.30 A.M. and 07.00 P.M.), with the total food amounting to about 2.5% of the horse’s body weight (forage:concentrate ratio 70:30); water was available ad libitum. The ration was similar to that usually administered by the owners. All horses were subjected to a 1660 m race simulation at “La Favorita” racetrack (Palermo, Sicily, Italy). The weekly training protocol is presented in Table 1.

### 2.2. Environmental Conditions

Thermal and hygrometric recordings were captured throughout this study using a probe capable of recording several parameters, with high precision and reading resolution. From the values obtained for mean temperature and relative humidity, it was possible to obtain the temperature–humidity index (THI) values. The THI was used as an indicator of thermal comfort for equine species, as previously validated, and suggested as the normal range in this animal species [12]. The THI was calculated using the following formula [12]:

THI [°C] = (0.8 × T°ambient) + {[(Relative humidity/100) × (T°ambient − 14.4)] + 46.4}.

### 2.3. Physiological Parameter Measurement, Blood Sampling, and Analysis

Data regarding respiratory rate (RR), heart rate (HR), and rectal temperature (RT) were recorded from each horse by the same operator. This was conducted while at rest in their box at 7:30 A.M. before the exercise (Pre) and within 5 min after the end of the exercise (Post), during the experimental period (May, June, and July). Specifically, the RR was determined by the observation of the movement of the horse’s flank, and a cycle of one rise and one fall of the flank constituted one breath. The HR was taken with a stethoscope. The RT was measured by means of a digital thermometer (HI92704, Hanna Instruments, Bedfordshire, UK), inserted 15 cm into the rectum. From all horses, blood samples were collected by jugular venipuncture and placed into 2 mL vacutainer tubes containing ethylenediaminetetraacetic acid (EDTA) and into 8 mL vacutainer tubes with cloth activator (Terumo Co., Tokyo, Japan). All samples were taken in the stables at rest before exercise (Pre) and within 5 min after the end of exercise (Post), after recording physiological parameters during the months of May, June, and July. Immediately after collection, the blood samples were transported for laboratory analysis. EDTA blood samples were analyzed for red blood cell count (RBC), hemoglobin concentration (Hb), hematocrit (Hct), mean corpuscular volume (MCV), mean corpuscular hemoglobin (MCH), mean corpuscular hemoglobin concentrations (MCHC), platelet count (Plt), and white blood cell count (WBC) [7]. The blood samples collected into cloth activator tubes were centrifuged (3000 rpm for 30 min) to obtain serum. The serum was then analyzed for the concentrations of cortisol, total proteins, albumin, and globulin fractions, as previously validated and described regarding horses [7].

### 2.4. Statistical Analysis

The Shapiro–Wilk normality test was applied to verify the normal distribution of the data. The data were normally distributed with a homogeneity of variance (*p* > 0.05) and expressed as mean values ± standard error of the mean (SEM). The two-way Analysis of Variance (ANOVA) was applied to assess the significant effect of the experimental conditions (exercise and months) on studied parameters. The Bonferroni post hoc comparison was applied when significant differences were found. The *p*-values < 0.05 were considered statistically significant. Prism software v. 9.00 (GraphPad Software Ltd., San Diego, CA, USA, 2020) was used for statistical analysis.

## 3. Results

**Figure 1:**
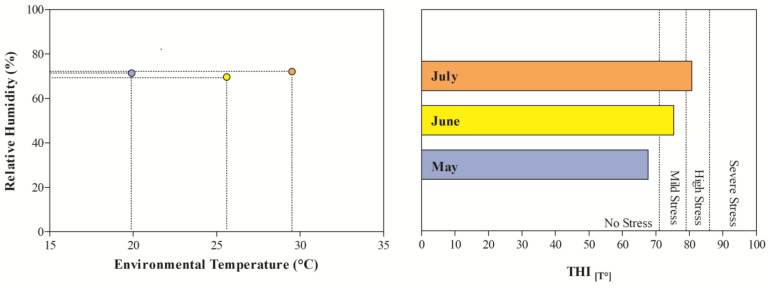
Environmental variable (i.e., relative humidity, temperature, temperature–humidity index (THI)) values recorded during the monitoring period.

**Figure 2:**
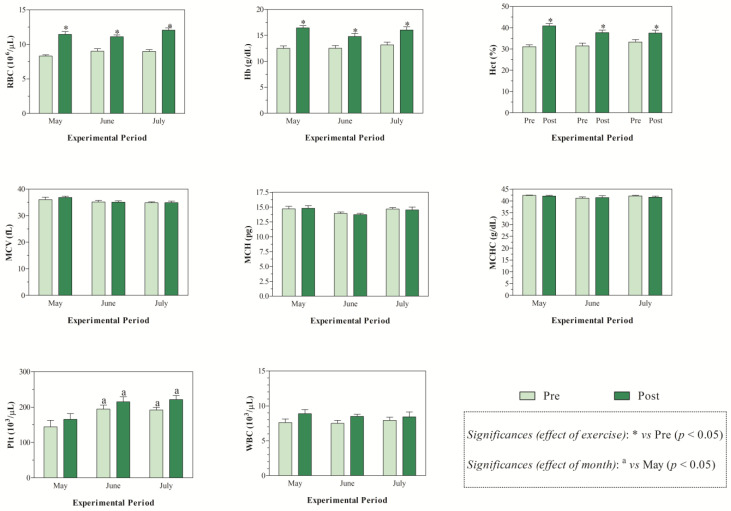
Mean values ± standard error of the mean (±SEM) of hematological parameters (i.e., red blood cell (RBC), hemoglobin (Hb), hematocrit (Hct), mean corpuscular volume (MCV), mean corpuscular hemoglobin (MCH), mean corpuscular hemoglobin concentrations (MCHC), platelet (Plt), and white blood cell (WBC)) measured in Standardbred horses before exercise (Pre) and within 5 min after the end of exercise (Post) during experimental period (May, June, and July).

**Figure 3:**
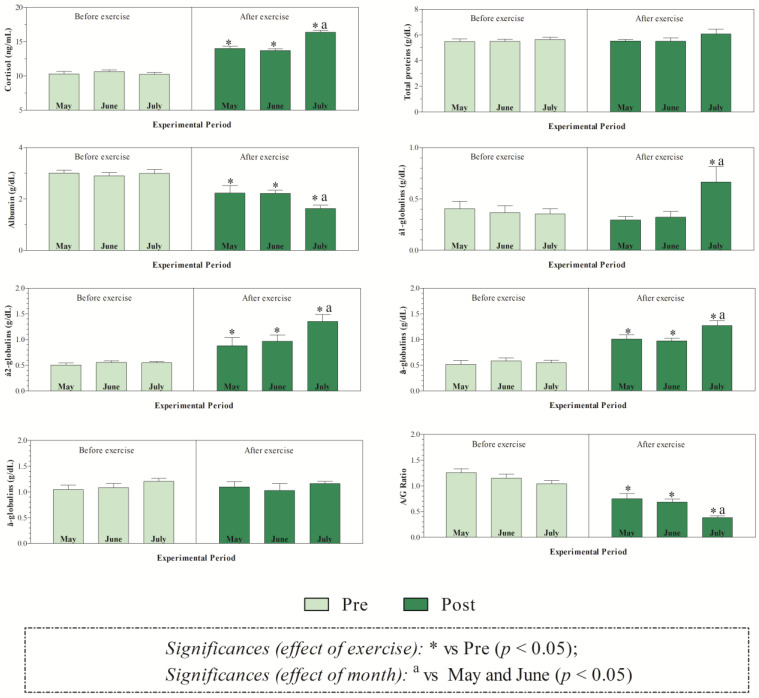
Mean values ± standard error of the mean (±SEM) of serum cortisol, total proteins, albumin, a negative acute phase protein, α1-, α2-, β-globulins known to include both negative and positive acute phase proteins, γ-globulins, and A/G ratio measured in Standardbred horses before exercise (Pre) and within 5 min after the end of exercise (Post) during experimental period (May, June, and July).

**Figure 4:**
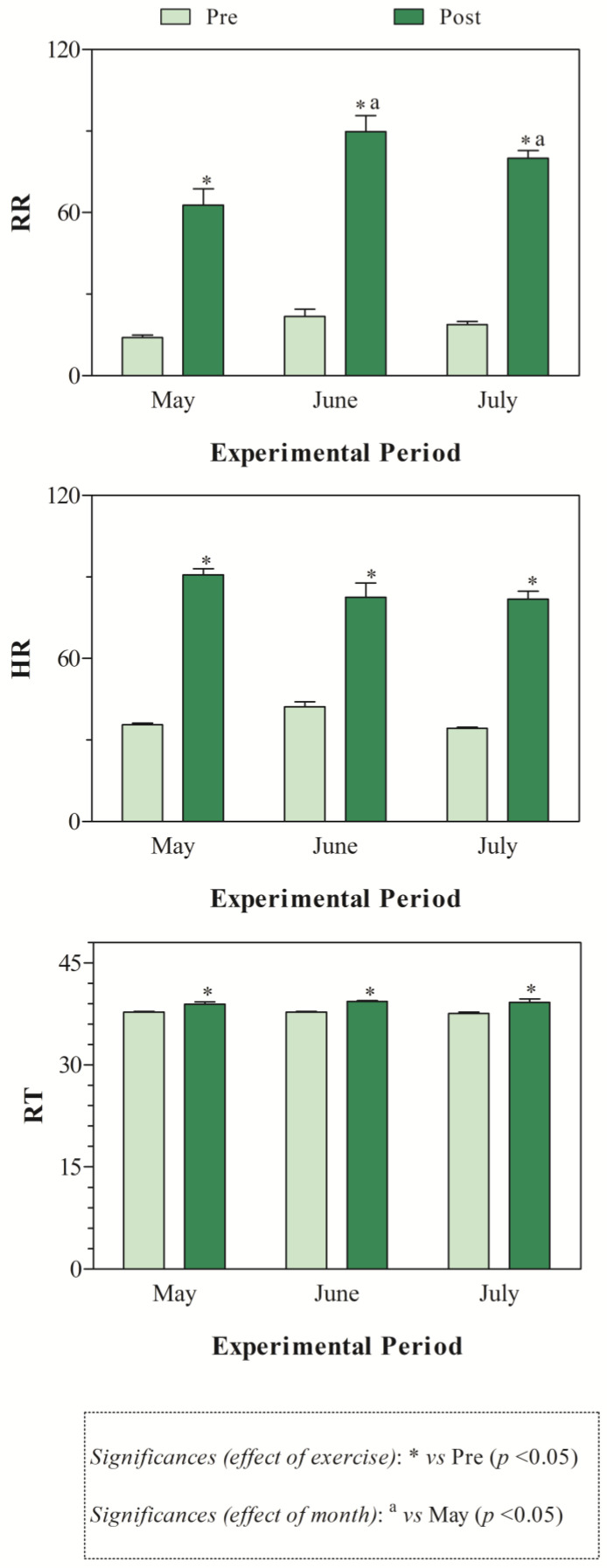
Mean values ± standard error of the mean (±SEM) of physiological parameters (i.e., heart rate, HR; respiratory rate, RR; rectal temperature, RT) measured in Standardbred horses before the exercise (Pre) and within 5 min after the end of the exercise (Post) during experimental period (May, June, and July).

The THI values recorded during the months of investigations indicated mild stress in June and high stress in July (Figure 1). The application of two-way ANOVA showed the significant effect of exercise (*p* < 0.05) on the values of RBC, Hb, and Hct. Specifically, as shown in Figure 2, the direct erythrocyte indices statistically increased after exercise compared with the values recorded at rest (*p* < 0.05). Moreover, higher serum concentrations of cortisol were found after exercise than at rest in May, June, and July (*p* < 0.05, Figure 3). Regarding serum protein fractions, lower serum albumin and A/G ratio values were found after exercise than at rest in May, June, and July (*p* < 0.05, Figure 3), higher α1-globulin values were found after exercise than at rest in July (*p* < 0.05, Figure 3), and higher α2- and β-globulin values were obtained after exercise than at rest in May, June, and July (*p* < 0.05, Figure 3). Regarding the physiological parameters investigated herein, the values of HR, RR, and RT statistically increased after exercise compared with the values recorded at rest in May, June, and July (*p* < 0.05, Figure 4). Higher PLT values were found in June and July than in May, both at rest and after exercise (*p* < 0.05, Figure 2). Moreover, after exercise, higher serum values of cortisol, α1-, α2-, and β-globulins, and lower serum values of albumin and A/G ratio were recorded in July than in May and June (*p* < 0.05, Figure 3). The values of RR recorded in horses after exercise were higher in June and July than in May (*p* < 0.05, Figure 4).

## 4. Discussion

Physical exercise is widely recognized as a stressful stimulus disturbing an animal’s homeostasis. Following a stressful stimulus, there is activation of the hypothalamic-pituitary-adrenal (HPA) axis and production of cortisol, which has the task of helping the body re-establish homeostasis [13,14]. However, cortisol alone is not always a definitive indicator of stress, especially in well-trained horses, and it may be modulated by training status, time of day, or other hormones. Overall, the results gathered in the present study highlighted the physiological responses of athletic horses to re-establish homeostasis following exercise. Specifically, exercise leads to increased values of direct erythrocyte indices (i.e., RBC, Hb, and Hct) compared to being at rest, likely due to the splenic contraction known to occur in response to physical effort [7]. These findings agree with previous investigations carried out on horses [2,15,16]. Regarding stress and inflammatory indices, a higher serum concentration of cortisol was found after exercise than at rest throughout the monitoring period, and higher α1-, α2-, and β-globulin values, as well as lower serum albumin and A/G ratio values, were found after exercise than at rest in May, June, and July. The globulins affected by exercise were those referable to acute phase proteins (i.e., albumin is a negative APP, the α- and β-globulin fractions include both positive and negative APPs, including haptoglobin, C-reactive protein, serum amyloid A, ceruloplasmin, fibrinogen, alpha 1-acid glycoprotein, complement proteins, transferrin), suggesting that horses investigated herein were stressed and in an inflammatory state following exercise. Contrariwise, horses subjected to physical effort were not dehydrated, as highlighted by unchanged serum total proteins found after exercise. Noteworthy, though the parameters signifying a stress and inflammatory response showed a change following physical exercise, the magnitude of the observed variations does not appear high enough to be of clinical relevance. The observed changes would seem to be linked to the physiological adjustments that the animal undergoes in response to exercise, with the ultimate goal of restoring homeostasis. The horses enrolled in the present study were well trained and accustomed to the type of work herein investigated. Indeed, enrolled horses were trained for racing and had previously participated in races. It has been suggested that experienced athlete horses have lower cortisol concentrations than inexperienced ones [17,18,19]. However, it has also been argued that cortisol may not reflect other axes of hormonal response (e.g., adrenal/testicular) [17]. Notably, the testosterone-to-cortisol (T:C) ratio is commonly regarded as a more robust indicator of physiological stress and anabolic/catabolic balance in both human and equine athletes [17,18]. For the physiological variables assessed herein, it is expected that a horse adapted to work should show changes in response to exercise and an ability to cope with these changes, shown by the recovery of basal values within a short period of time. The values of HR, RR, and RT increased after exercise compared with the values recorded at rest conditions in May, June, and July. For HR, this response to adrenergic stimulus over the heart was expected [3], and was the equivalent to HR at submaximal work [20], because the HR tends to positively correlate to the level of effort required for work [21]. The significant increase in RR is probably due to an increase in the demand for oxygen associated with the work performed and the need for better alveolar ventilation to eliminate the carbon dioxide produced [3]. During exercise, ventilation increases in response to increased metabolic demand, and the magnitude of this increase depends on the intensity and duration of exercise [22]. Though a statistical change related to exercise was found for the RT, the values of this parameter fall within the physiological range, suggesting a good thermoregulatory response of the investigated horses. Notably, the use of infrared thermography as a more sensitive, non-invasive tool would be more appropriate, as it can detect local heat buildup or vascular changes [23]. During exercise of moderate intensity, the equine should be able to dissipate heat, keeping temperature near basal values within 10 min after the end of exercise [24]. In this study, the recording of physiological parameters, including RT, was performed within 5 min from the end of physical effort; therefore, horses could have shown a complete recovery to basal HR, RR, and RT values, whether the determination was carried out in a longer time frame than the end of the exercise, as previously found [19]. When considering the effect of months on investigated parameters, the results gathered in this study showed that, among the physiological parameters investigated in this study, only the RR showed month-related changes in horses, displaying higher values in June and July than in May, and only after exercise. Environmental factors, such as ambient temperatures and relative humidity, also contribute substantially to a change in respiratory rate. Since the increase in RR values observed herein was statistically significant, only after exercise and not under resting conditions in the warmest month, it could be hypothesized that the investigated horses were quite adapted to the environmental conditions, as indicated by the absence of month-related changes in HR and RT values. The higher ambient temperatures, together with the values of the relative humidity recorded in June and July compared to May, would have emphasized the physiological effect that exercise has on RR.

A study carried out on reining horses acclimatized or non-acclimatized to heat stress showed that the difference in RR of horses at rest may have been caused by the slow reaction of the vasculature during increasing cutaneous blood flow of horses in the hot environment, thus not meeting the body’s requirement to maintain core temperature and subsequently removing heat via the respiratory tract [19]. Though the lactate concentration was not investigated in the current study, it could be hypothesized that the increase in RR in the horses herein investigated may be due to increased lactate concentration with a consequent pH decrease likely to occur after the exercise [19]. Specifically, an increased hydrogen ion concentration in the circulation and the extracellular fluid results in an increased alveolar ventilation and therefore increased RR [19]. Furthermore, the results gathered in this study showed no difference related to month in the hematological indices, with the exception of PLTs, which showed higher values, both at rest and after exercise, in June and July than in May. Moreover, after exercise, higher serum values of cortisol, α1-, α2-, and β-globulins and lower serum values of albumin and A/G ratio were recorded in July compared to in May and June. The thermoneutral zone for horses ranges from 5 to 25 °C [25]. When ambient temperatures rise above this range, the animal must expend energy to dissipate excess heat. Conversely, when temperatures fall below this zone, the horse needs to increase metabolic heat production to maintain its core body temperature [6,26]. The results observed in this study suggest that Standardbred horses experience stress and inflammation when exercising in warmer seasons. It is well known that under stressful stimuli, the sympathetic nervous system releases adrenaline and noradrenaline, which can stimulate the release of PLTs from the bone marrow and spleen, resulting in increased circulating PLT values. Noteworthy, it is well established that stress can impair an animal’s immune function, making it more susceptible to diseases and negatively impacting both its welfare and performance [13,14]; therefore, the highest cortisol values found during warmer months, together with higher acute-phase proteins, seems to suggest a negative condition for animals that are more prone to the onset of pathological conditions. This interpretation appears to align with the THI values recorded over the three-month study period. In particular, the index indicated moderate heat stress in June and severe stress conditions in July during the selected monitoring months of May, June, and July. However, the magnitude of the variations observed in these biomarkers does not appear high enough to be of clinical relevance.

The current study has several limitations: (i) the sample size (*n* = 12) of investigated horses, which could be relatively small and could limit statistical power and generalizability; (ii) the post-exercise measurement timing (within 5 min) may not capture delayed peaks in certain investigated biomarkers; (iii) due to the quantity of serum, which was not enough, more parameters such as lactate, testosterone, or other hormonal/metabolic markers were not assessed in the current study.

## 5. Conclusions

Awareness of the functional adaptation of horses to a stressful circumstance, such as physical exercise, is of paramount importance for the monitoring of their health status. The ability of an animal to elicit a suitable response to a stimulus that provokes a threat to its homeostasis is decisive to its survival. The current study provided useful information on the physiological response and coping capacity of Standardbred horses in Sicily during warmer months. According to cortisol and electrophoretic protein pattern changes observed in horses after exercise, it could be speculated that the stress and inflammatory responses of the horses, investigated herein, are activated. The magnitude of the variations observed in these indices does not appear high enough to be of clinical relevance, but rather, they would seem to be linked to the physiological adjustments that the animal undergoes in response to the exercise, with the ultimate goal of restoring homeostasis. Enrolled animals were well-trained horses; therefore, it is likely that horses have become accustomed to this type of work. The stress and inflammatory response, as well as the RR of horses investigated herein, proved to be more marked in July when THI highlighted a high heat stress condition. Therefore, despite the effort of trotting does not appear to constitute a challenge for the physiological adjustments of the well-trained horses following exercise, a more careful assessment of the hydration and general health of these animals during warmer months is encouraged.

## Figures and Tables

**Table 1:** Tabular representation of the work performed in weekly training routines.

Gait		1st, 2nd, 4th, and 5th Day	3rd Day	6th Day
Walk	Speed	100 m/min	100 m/min	100 m/min
	Duration	10 min	15 min	15 min
Trot	Speed	350 m/min	670 m/min	Simulation race 1660 m
	Duration	25 min	6 min	
Walk	Speed	100 m/min	100 m/min	100 m/min
	Duration	10 min	15 min	15 min

## Data Availability

Data are contained within the article.
